# Sociodemographic and Psychological Correlates of Sun Protection Behaviors among Outdoor Workers: A Review

**DOI:** 10.1155/2013/453174

**Published:** 2013-10-22

**Authors:** Vinayak K. Nahar, M. Allison Ford, Jeffrey S. Hallam, Martha A. Bass, Michael A. Vice

**Affiliations:** ^1^Department of Health, Exercise Science & Recreation Management, The University of Mississippi, 215 Turner Center, P.O. Box 1848, University, MS 38677, USA; ^2^Department of Social and Behavioral Sciences, College of Public Health, Kent State University, 750 Hilltop Drive, Kent, OH 44242, USA

## Abstract

Outdoor workers are at a higher risk for developing skin cancer due to their increased sun exposure. The primary objective of this review was to synthesize the current research literature that addresses sociodemographic and psychological factors related to sun protection behaviors in outdoor workers. Two additional purposes were to provide an overview of sun exposure and describe sun protection behaviors of outdoor workers. To identify the studies for this review, a methodical search was performed in the PubMed, PsycInfo, MEDLINE, and ERIC databases. Fifteen studies met the review criteria. Despite regular and prolonged sun exposure, many outdoor workers fail to engage in sufficient sun protection behaviors. Correlates of outdoor workers' sun protection behaviors include being female, older age, being white, personal skin cancer history, time (hours/years) spent at work, sun safety training, perceived prioritization of sun protection, concern about sun exposure, workplace support, families' expectations, and familial information giving. However, limited attention is given to designing theoretically grounded studies to identify factors to inform future research. There is a need to conduct research based on solid theoretical foundations that explains the relationships among the factors in this domain.

## 1. Introduction

Skin cancer is a significant public health problem in the US [1, 2]. Each year over 3.5 million cases of skin cancer are diagnosed, resulting in nearly 12,000 deaths [3]. Since sun exposure is the primary risk factor for all forms of skin cancer, nearly 90% of skin cancers are considered preventable [4, 5]. Recommended sun protection behaviors include using the shade, avoiding being outdoors during the hours of highest sun intensity (between 10:00 a.m. and 4:00 p.m.), and using sun protective clothing, hats, sunglasses, and sunscreen, preferably a sunscreen with a sun protection factor (SPF) not less than 30 [3].

All individuals are at risk for developing skin cancer; however, there are groups, most notably outdoor workers, who are more vulnerable to skin cancer compared to other populations [6, 7]. This is quite obvious considering the regular and considerable amount of time they spend exposed to solar ultraviolet radiation (UVR) during work—at least two to eight hours per day [8, 9]. In addition, it is documented that outdoor workers exposure to UVR is much higher than the recommended guidelines [8]. 

 There is substantial evidence to support the significant association between skin cancer and cumulative, as well as, intermittent sun exposure in outdoor workers [7, 10–13]. In addition, solar UVR dose received by outdoor workers is about six to eight times higher than indoor workers, and outdoor workers have a greater chance of being diagnosed with skin cancer [8, 14–17]. In addition, high incidence and mortality rates of skin cancer are found in occupational groups that work outdoors [18–20].

 Outdoor workers make up a sizable proportion of the work population that spreads across a wide range of jobs. According to the US Census Bureau, occupational groups that work outdoors represent more than eight percent of the total US national work force (over 9 million workers) [21]. These groups tend to have an ethnoracial majority of fair skinned individuals which is strongly linked with an elevated risk for skin cancer, primarily due to inherently low amounts of melanin present in the skin, the pigment responsible for skin color and protection against harmful UVR [21–24].

Furthermore, several epidemiological studies note that men are at a significantly higher skin cancer risk than women [25–27]. Existence of skin cancer development disparity between men and women is not a natural phenomenon but is due to greater percentage of men in outdoor occupations and the differences between men and women in skin protection behaviors and lifestyle choices [24, 26–31].

Despite the fact that sun protection behaviors are promoted, incidence rates of skin cancer in outdoor workers continue to be high. Prior research has addressed personal and behavioral factors, but no review published to date has examined the sociodemographic and psychological correlates of skin cancer and the sun protection practices of outdoor workers. Therefore, the primary objective of this review is to provide an overview of the available research that addresses sociodemographic and psychological factors associated with sun protection behaviors in outdoor workers. In addition, this review includes a description of sun exposure and sun protection practices of outdoor workers. 

## 2. Methods 

To identify the studies for the review, a methodical comprehensive computerized search was performed using PubMed, PsycInfo, MEDLINE, and ERIC databases. The terms “skin cancer,” “melanoma,” “sun protection,” “sun exposure,” “sun behavior,” and “skin cancer prevention in outdoor workers,” were searched as keywords or phrases. Additional searches were performed in the websites of the following organizations: the Centers for Disease Control and Prevention (CDC), the American Cancer Society (ACS), the American Academy of Dermatology (AAD), the Skin Cancer Foundation, and the Melanoma Foundation of Australia. Bibliographies of articles were also manually searched to identify pertinent articles that were not identified in the initial search.

The search was limited to studies published in English. In the last two decades, there was a surge in interest and research on the topic of skin cancer; therefore, the decision to review articles published from 1990 to the present was made to include seminal research conducted within the last 20 years.

Research studies specifically emphasizing outdoor workers' skin cancer or sun-protection-related knowledge, beliefs, behaviors, and attitudes towards sun safety were considered eligible for inclusion in the review. Articles were excluded if (1) the results of the article were not relevant to the aims of the review; (2) the article examined clinical issues or the effect of specific treatments or settings; (3) the article described sun protection behavior of different population groups along with outdoor workers; (4) the article was a duplicate, a conference abstract, an editorial, a case report, a letter, or commentary; or (5) the article was published in an online newspapers that is not peer reviewed. 

**Figure 1:**
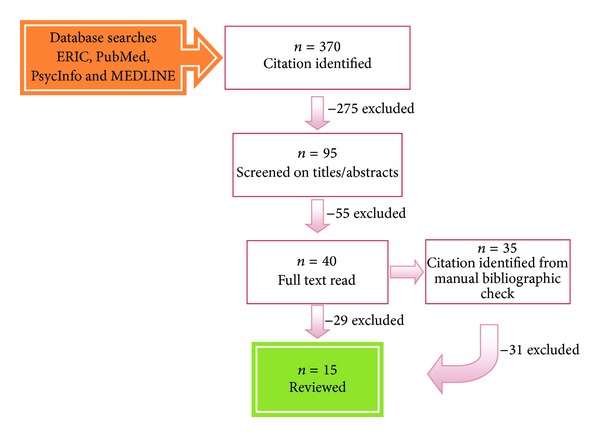
Flow chart of the literature search.

The electronic search identified 370 citations, with 275 citations being excluded on the basis of the inclusion criteria. Titles and abstracts of the remaining 95 citations were screened, and 55 more articles were eliminated. The remaining 40 articles were read to determine if they met the inclusion criteria. We identified 35 additional articles from the reference lists of the 40 articles. Of these 75 articles, 15 fully met the inclusion criteria and were included in this review. The summary of reviewed studies is provided in Table 1, and the process of the literature search is illustrated in Figure 1.

## 3. Results

### 3.1. Demographic

#### 3.1.1. Disparity between Men and Women

Of the five studies which reported sun protection behavior of both male and female outdoor workers, four studies documented differences between men and women in sun-related behaviors. Rosenman et al. (1995) found that female farmers in Michigan were more likely to practice some type of sun protection behaviors than male farmers [32]. Further support for gender differences was found in studies on postal workers. Results showed that female postal workers were more likely to wear sunscreen, whereas their male counterparts were more likely to wear a hat [6, 33]. Another important finding noted by Lewis et al. (2006) was that being female was the only common predictor of sunscreen use for both working and nonworking days [33]. Moreover, in a New Zealand study with a large sample of outdoor workers (*n* = 1,283), consistent with the previous studies, McCool et al. (2009) found that females were significantly more likely to wear sunscreen than males [34]. On the other hand, Stepanski and Mayer (1998) did not find a difference in UVR protection behaviors between male and female outdoor workers although this similarity in sun protective behaviors may result from clothing policies enforced by the companies [35].

#### 3.1.2. Age

There is evidence showing the relationship between age of outdoor workers and skin cancer prevention behaviors, although the study by Stepanski and Mayer (1998) demonstrates no correlation between age and sun protection behaviors [35]. However, Rosenman et al. (1995) show that increasing age influenced individuals to use protective measures against sun exposure [32]. Moreover, in-depth interviews conducted by Parrott et al. (1996) reveal that older participants were more willing to engage in sun protection practices than younger counterparts [37]. Supporting this, McCool and colleagues (2009) note that a greater likelihood of sunscreen use was related to being older [34]. In addition, Madgwick et al. (2011) find that age was positively correlated with wearing long-sleeved, loose fitting tops and trousers [44].

#### 3.1.3. Ethnicity

Ethnic background was recognized in the following studies as one of the factors related to outdoor workers sun protection behavior. Pichon et al. (2005) surveyed 2,660 participants (non-Latino White, Latino, Asian American, African American, and Pacific Islander) to compare sun-safety behaviors across ethnoracial groups employed as letters carriers at United States Postal Service (USPS) [6]. Results show that ethnicity was significantly associated with the use of sunscreen and sunglasses. Also, rates of sunscreen and sunglasses use in non-Latino White are significantly higher than the other four groups. Similar results for sunscreen use are echoed in a Lewis and colleagues (2006) study conducted one year later (i.e., sunscreen use at work is significantly associated with ethnicity and sunscreen use in non-Latino White postal workers was significantly higher than in Asians and African Americans postal workers) [33]. Therefore, ethnicity correlates with particular sun protection behaviors.

#### 3.1.4. Skin Type

Influence of skin type on sun protection behavior is investigated in many studies. Woolley et al. (2002) report a positive relationship between skin type and sun protective clothing use [29]. An encouraging result emerging from this study is that outdoor workers with more vulnerable skin types avoided the sun between 10:00 a.m. and 2:00 p.m.. Pichon et al. (2005) and Lewis et al. (2006) show increased sunscreen use and hat use with greater sun sensitivity [6, 33]. On the contrary, there are studies that yield no significant association between skin type and sun protection clothing use [34, 41]. However, Salas et al. (2005) speculate that there is no relationship because the use of sun protection clothing by outdoor workers is not to protect their skin but to protect themselves from occupational hazards such as handling pesticides or thorny branches [41]. Additionally, in McCool et al.'s (2009) study, researchers examined only sunscreen use behavior, whereas studies that show an association investigate more than one sun protection behavior [34]. After taking all the results and limitations of these studies into consideration, it is concluded that sensitive skin type plays a role in the sun protection behavior of outdoor workers. 

#### 3.1.5. Education

Two studies were found that examined the relationship between education and sun protection behavior. The results are equivocal. Rosenman et al. (1995) report that increased education in farmers did not affect the likelihood of using sun protection [32]. Moreover, findings from a more recent study indicate that outdoor workers with higher education were significantly more likely to use sunscreen than outdoor workers with lower education [34]. The scarcity and ambiguity of data on the impact of education in outdoor workers makes it difficult to draw any firm conclusion.

#### 3.1.6. Income

To our knowledge, only one study documents the influence of income on sun protection behaviors [32]. Results show that increased income in female farmers appeared to increase the likelihood of using sun protection presumably because money is required for the purchase of sun protection modalities; whereas, male farmers show no increase in the likelihood of using sun protection with an increased income. 

#### 3.1.7. Time (Hours/Years) Spent at Work

The amount of time (hours/years) that outdoor workers spend at work is found to be related to sun protection behavior. Lewis et al. (2006) show that the use of occupational sunscreen and hats in postal carriers is positively associated with hours worked outdoors [33]. Also, Madgwick et al. (2011) report that the more time construction workers spend outdoors, the more likely those construction workers will wear wide-brimmed hats [44]. In terms of years, Salas and colleagues (2005) note that participants who use higher levels of sun protective clothing worked as farm workers a significantly longer period of time than the participants who reported lower levels of protection [41]. 

#### 3.1.8. Personal History of Skin Cancer

The evidence of the relationship between personal history of skin cancer and sun protection behaviors in outdoor workers is clearly seen in the study of Woolley et al. (2002) in which solar protection (77.4% wore wide-brimmed hats, 52% wore long-sleeved shirts, and 50% wore sunscreen when out for significant amount of time) of male outdoor workers with previously removed nonmelanoma skin cancer was considerably higher than solar protection of outdoor workers in other studies [29]. This reflects the finding of a prior study that documented personal history of skin cancer increased the likelihood of sun protective measures use in farmers and their spouses [32]. 

#### 3.1.9. Family History of Skin Cancer

There is not sufficient evidence to support the influence of family history of skin cancer on sun protection behavior amongst outdoor workers. Participants of Rosenman et al.'s (1995) study did not show increased use of sun protection measure against sun if they had a family member or friend with skin cancer history [32]. Also, Stepanski and Mayer (1998) noted that UVR behavior score did not vary between participants with and without a family history of skin cancer [35]. Lewis et al. (2006) report having a family history of skin cancer as being significantly associated with engaging in sunscreen use, whereas no association was reported with occupational hats use [33]. Unfortunately, with only one study that somewhat supports the association between family history of skin cancer and outdoor workers sun protection behavior, any conclusion is speculative at best. 

#### 3.1.10. Sun Safety Training

The results of Madgwick et al.'s (2011) study show a positive association between receipt of sun safety training and sun protective behaviors including use of sunscreen [44].

### 3.2. Psychological

#### 3.2.1. Knowledge

Of the research reviewed on sun protection behavior, one factor assessed in several studies is knowledge related to skin cancer or melanoma. A large number of studies reported that there appears to be a reasonable level of knowledge about the skin cancer. Wisconsin dairy farm workers report an average score of 70% correct on knowledge questions about skin cancer [36]. Also, 83% of Georgia farmers report having knowledge that the level of SPF in sun block or sunscreen should be 15 or higher and 90% indicate that melanoma is the most dangerous type of skin cancer [37]. In an Australian study, most of the outdoor construction workers report a high level of knowledge about skin cancer risk (94%), common areas of body to cover with sunscreen (82%), and use of sunglasses (85%) [39]. Moreover, the researchers of these studies report that this knowledge is not translated into sun protection behaviors; therefore, actual engagement in skin cancer prevention practices was poor. These findings further support the finding of Hammond et al. (2008) who show that sun protection practices are related to personal factors such as perceived susceptibility of developing skin cancer and perceived workplace support but not to knowledge about skin cancer and prevention [42]. However, there is an inconsistency in the literature with regard to association between knowledge and skin cancer prevention. Studies yielded conflicting data, for example, Parrott and Lemieux (2003) find skin cancer knowledge of farmers positively associated with use of sunscreen, long-sleeved shirts, and sun protective hats [40]. Another example is McCool et al.'s (2009) who describe sunscreen use as strongly related to perceived knowledge about skin cancer [34]. 

#### 3.2.2. Perceived Susceptibility

Of the research studies reviewed, only two studies examined the role of perceived susceptibility to skin cancer in determining sun protection behavior. Marlenga (1995) indicates that participants perceived a susceptibility to skin cancer; however, they did not use sun protection methods [36]. In contrast, Hammond and coworkers (2008) report that increased perceived susceptibility to skin cancer is one of the factors that increased the likelihood of using sun protection in outdoor workers [42]. Based on contradictory findings, it is not possible to suggest that sun protection behavior is associated with perceived susceptibility. 

#### 3.2.3. Perceived Barriers

A considerable amount of perceived barriers to sun protection are recognized in the reviewed studies. These include difficulty in remembering to use [29, 36, 38], amount of time [37], inconvenience or uncomfort to use [29, 36–38], not worrying about sun exposure [38], and perceived physical attractiveness of suntan [36, 38, 39, 44]. Furthermore, results of a study conducted by Marlenga (1995) reveal that, of all the addressed Health Belief Model (HBM) variables (except self-efficacy), perceived barriers was the only important predictor of whether farmers protect their skin from sun exposure [36]. 

#### 3.2.4. Suntan Attitude

Two studies report an association between suntan attitudes and sun protection behaviors although findings were inconsistent. Hammond et al.'s (2008) study results suggested that positive suntan attitude in outdoor workers tended to reduce sun protection use [42], although findings from McCool et al.'s (2009) study suggest no significant association [34].

#### 3.2.5. Perceived Priority

 McCool et al. (2009) suggest that perceived priority of sun protection at work was significantly associated with use of sunscreen [34].

#### 3.2.6. Concern

The results of McCool et al.'s (2009) study also suggested significant association between higher concern about sun exposure at work and sunscreen use [34].

### 3.3. Social

#### 3.3.1. Workplace Support

In the recent years, researchers have investigated the association between workplace support and sun protection behavior in outdoor workers. McCool et al. (2009) report a positive association between workplace support and sun protection practice [34]. The findings of this study corroborate the results of a previous study [42]. This association is strong and persistent.

#### 3.3.2. Familial Expectations and Information Giving

Parrott and Lemieux (2003) report that familial expectations and information giving was positively correlated with sun protection behaviors [40].

### 3.4. Sun Exposure

A Canadian national survey on sun exposure and protective behaviors reports that 70% of participants who worked outdoors experienced more than two hours of sun exposure during an average working day [38]. Sun exposure of construction workers in Britain was estimated at 6.6 hours per day [44]. In the US, construction workers, transportation workers, and letter carriers spent an average of 7.94, 6.95, and 5.11 hours, respectively, working outdoors [35]. Moreover, surveys of larger samples of postal workers in Southern California report receiving an average of 4 hours of sun exposure on workday [6]. Similarly, Wisconsin dairy farmers report being outdoors 4.15 hours daily [36]. Lifeguards in Austin, Phoenix, Omaha, and Portland recount spending an average of 4.29 hours a day in the sun [43].

Additionally, farmers report an average of between 14 and 43 years of farming experience [36, 40, 41]. Postal workers indicate an average of 12 years of prolonged occupational sun exposure history [6].

### 3.5. Sun Protection Behaviors

Most of the studies examined the use of at least two of the following sun protection measures in combination: wearing a hat, sunscreen application, wearing sunglasses, wearing protective clothing, and staying in the shade or otherwise limiting exposure to sun during the midday hour. Field observations studies conducted on transportation workers, construction workers, and postal workers revealed that 50.4% of the workers adequately protected their skin from the sun [35]. However, among Wisconsin dairy farmers, only 7% report that they frequently or always wore long-sleeved shirts, 13% report frequently or always wearing wide-brimmed hat, and 8% report frequently or always using sunscreen [36]. Among Californian farmworkers, few report frequently or always wearing a wide-brimmed hat (6%), using sunscreen (1.6%), and wearing sunglasses (2.6%) when working outside in the sun for more than 15 minutes. On the other hand, a considerable number of California farmworkers report frequently or always wearing a shirt with long sleeves (89.7%) [41]. Farmers, road workers, construction workers, and other outdoor workers in Georgia describe less use of adequate sun protection. Although 86% wore long pants and 74% wore sunglasses, only 5% report wearing wide-brimmed hats or caps with flaps and 5% report wearing long-sleeved shirts [37]. 

The sun protection behavior patterns of outdoor workers observed in the US are similar to those in other countries. Studies note that many outdoor workers fail to adequately engage in sun protective practices. Participants in Canadian research on outdoor workers conclude the following sun safety practices: wearing protective clothing (60%), wearing hats (58%), avoiding the sun (38%), and using sunscreen (between 18% and 23%) [38]. In Britain, the most commonly used primary prevention strategies include using sunscreen (60%), wearing long-sleeved tops or trousers (51%), and wearing sunglasses (44%), with fewer participants reporting that they wore wide-brimmed hats (23%), and were using shade or otherwise limiting exposure to sun (between 19% and 23%) [44]. Likewise, construction workers in a study carried out in Australia report frequently or always wearing sunglasses (61%), using wide-brimmed hats (54%), using sunscreen (34%), wearing long-sleeved shirts (11%), and using shade (5%) [39].

Furthermore, studies identify the UVR exposure pattern of outdoor workers during workdays and days off. Among male outdoor workers in Australia, 51% of the participants experience over six hours of sun exposure on an average working day and 76% spent over two hours in the sun on the average weekend day or day off [29]. With regard to sun protection behavior, 43.6% and 77.2% usually wear long-sleeved shirts and wear a wide-brimmed hat, respectively. Mail carriers report reasonably similar amounts of sun exposure for working (3.9 hours) when compared to nonworking (3.3 hours) days, although reported use of protective measures for nonworking days was considerably lower [33]. On working days, 24% of mail carriers recount wearing a hat versus only 4% wearing a hat on nonworking days and 25% recount using sunscreen on working days and 12% on nonworking days [33]. 

## 4. Discussion

Outdoor workers constitute an important target group, who are susceptible to developing skin cancer, given the considerable amount of hours they spend outdoors on workdays and days off. This intense UVR exposure is experienced by outdoor workers for prolonged periods throughout their lives, since they tend to spend several years in outdoor occupations. Although receiving high UVR exposure on regular basis, overall data show that a majority of outdoor workers did not adequately protect themselves from sun exposure. 

The findings of this review suggest that there are several factors that correlate with outdoor workers' sun protection behaviors. These correlates include being female, older age, being White, a personal history of skin cancer, time (hours/years) spent at work, sun safety training, perceived priority of sun protection, concern about sun exposure, workplace support, families' expectations, and familial information giving. On the other hand, factors that appear to be related to lower levels of sun protection behavior include being male, younger age, and reporting perceived barriers. 

With regard to sun protection behavior, findings were sparse and inconsistent regarding the relationship of factors such as skin type, education, income, perceived susceptibility, and suntan attitude. Therefore, considerably more research work is required to determine potential importance of these factors and before any conclusion is drawn regarding the relationship of these factors to engaging in sun protection practices.

The relationship of family history of skin cancer with sun protection behavior is likewise difficult to assess because in the research reviewed, this item was assessed through use of a single dichotomous question that did not define first-degree relatives (i.e., parent, sibling, or child). Additional research that explores this issue through a more specifically worded question might provide more accurate and useful results. 

Knowledge of skin cancer is widely studied for its relationship to sun protection behavior, and research on this construct continues to yield inconsistent results. This may be accounted for by differences in measures, methods, and analysis. However, in general, outdoor workers report that they are knowledgeable about skin cancer, but many do not engage in adequate sun protection behaviors. Therefore, it may be that knowledge alone is not enough to lead to sun protection behaviors. It is possible that other cognitive factors or combinations of factors are influencing the adoption of sun protective behaviors. At this time, however, little is known about psychological factors that explain why outdoor workers, despite a high level of knowledge about skin cancer, choose not to practice sun protection.

Only two of the identified articles described research that had a theoretical foundation. One study was based on constructs of the HBM [36, 45]. Perceived barriers were found to be the single predictor that explained why farmers did not engage in sun protection practice which led the author to suggest that the utility of HBM with Wisconsin dairy farmers is questionable. It should be noted, however, that this research did not utilize the revised version of HBM which includes the construct of self-efficacy [46]. This leads one to the following question: what would the results be if the author had included self-efficacy in the study?

The other theoretically grounded study was based on social cognitive theory (SCT) [37]. The purpose of the study was to assess use of constructs of SCT to identify personal determinants of farmers' skin cancer and prevention behavior and environmental influences that might either facilitate or inhibit the impact of skin cancer prevention campaigns directed at farmers. Unfortunately this research was conducted as a pilot study with an instrument that had no established psychometric properties, so both reliability and validity of these findings are uncertain. 

No study focused exclusively on the relationship between sun protection self-efficacy and sun protection behaviors among outdoor workers. Self-efficacy reflects the confidence an individual has in his or her ability to successfully perform a behavior in order to achieve a desired outcome [47]. A self-efficacy-based intervention demonstrated some success in improving the responsiveness of young females to health information regarding skin cancer and aging effects of the sun [48]. Therefore, this construct merits further study in the context of outdoor workers.

 Generalizing the results of these multiple studies is made difficult due to the range of occupations that comprise outdoor workers. Farmers, construction workers, and postal workers are the most frequent targets of research on sun protection behaviors. However, specific behaviors may differ among outdoor workers due to specific job types, proportion of males or females in occupations, and ethnicity/race [21, 41]. In addition, few studies investigated participants in a variety of occupations, and most of the studies did not examine the differences in sun protection behaviors between the subgroups of the samples. Assessing sun protection behaviors of subgroups in an outdoor worker population will be useful in designing or tailoring effective and specific group-focused sun protection intervention which addresses the specific sun protection needs of each specific group. 

## 5. Limitations

 This review has several shortcomings that need to be addressed. First, this review is subject to publication bias because the authors limited their review to studies published in English. Second, reviewed studies had cross-sectional designs, and therefore causation cannot be determined. Third, since the data gathered in the studies were collected through self-report, the influence of social desirability and recall bias in the results cannot be discounted. Finally, the majority of the reviewed studies did not report the validity and reliability of the survey instruments used to collect the data, and therefore the results are suspect.

## 6. Conclusion

This literature review provides an assessment on a variety of sociodemographic and psychological factors that are related to the likelihood of outdoor workers adopting sun protection behaviors. Unfortunately, few theoretically grounded studies, which may have greater potential to identify factors or to generate predictions, have been published. The studies identified for this review that had greater theoretical emphasis were weakened by methodological issues. Without further assessment, it is difficult to determine whether the existing health behavior theories are useful in predicting sun protection behavior in outdoor workers. There is a need to conduct a study based on solid theoretical foundations that attempts to provide a potential and systematic explanation of relationships of factors in this domain. A deeper understanding of factors influencing sun protection practices could serve as a base for future studies and preventive interventions.

## Figures and Tables

**Table 1:** A Summary of the reviewed studies.

Author, date	Study design, methods	Population, sample size (*n*)	Average sun exposure and occupational years	Sun protection behaviors	Barriers to sun protection	Correlates of sun protection behavior	Limitations
Marlenga [36]	Cross-sectional Self-administered mail surveys	Dairy farmers, 100% male, *n* = 202	4.15 hours/day 42.95 years	Long pants (90%), wide-brimmed hat (13%), sunscreen (8%), and long-sleeved shirts (7%)	Too hot to wear long-sleeved shirt (88%), tan looks attractive (>50%), and I forgot to wear sunscreen (45%)	*Psychological: *perceived barriers	Results were not generalizable to all farmers

Rosenman et al. [32]	Cross-sectional Self-administered mail survey	Farmers, *n* = 1,342	Not reported	65% of the women and 43% of the men were very likely to practice some type of sun protection	Not reported	*Demographic: *female gender, increased age, higher personal history of skin cancer, and greater income among females	Results were not generalizable to all rural populations Superficial data on potential confounders

Parrott et al.[37]	Cross-sectional pilot Intercept survey, field observations, and in-depth interviews	Intercept survey: 155 farmers, 100% White Fields observations: 49 farmers, 41 construction workers, 39 road workers, and 15 other outdoor workers In-depth interview: 9 farmers	Not reported	65% did not wear long-sleeved shirts, 49% did not apply sunscreen, and 43% did not wear wide-brimmed hats	Amount of time needed to put on long-sleeved shirt (30%), wide-brimmed hats are uncomfortable (21%), and sunscreen is messy to apply (11%)	*Demographic: *increased age	Not reported

Stepanski and Mayer [35]	Cross-sectional field observation, and self-administered survey	Construction workers, Caltransworkers, and mail carriers 80.1% males and 61.0% White, *n* = 240 (survey data) *n* = 312 (observation data)	5.11–7.94 hours/work day	50.4% reported sufficient use of sun protection (observational data)	Not reported	Not found	Observational data did not represent typical sun protection behavior Convenience sample Recall, social desirability, and self-selection bias Study design

Shoveller et al. [38]	Cross-sectional Telephone survey	General outdoor workers, 80% male, *n* = 546	>2 hours/work day (70%)	Sun protective clothing (60%), hat (58%), sunscreen on face (23%), and sunscreen on body (18%)	I forgot (61%), inconvenient (54%), liked tanned skin (38%), and not worried about UVR exposure (34%)	Not reported	No information was provided about types of hats worn and lengths of sleeves on shirts

Cioffi et al. [39]	Cross-sectional Self-administered survey	Construction workers, 97.8% males, *n* = 142	Not reported	Sunglasses (61%), wide-brimmed hat (54%), sunscreen (34%), long-sleeved shirt (11%), and use of shade (5%)	Perceived tan is attractive (72%) and healthy (44%)	Not reported	Validity and reliability of instrument was not tested Convenience sample

Woolley et al. [29]	Cross-sectional Self-administered mail survey	General outdoor workers, 100% males, *n* = 300	>50% of mainly outdoor workers spent >6 hours/work day	Wide-brimmed hat (77.2%) and long-sleeved shirts (43.6%)	Not get around to putting it on (24%), inconvenience (22%), and forget to bring it along (21%)	*Demographic: *sun sensitive skin type and personal history of skin cancer	Recall bias Self-selection bias Low response rate

Parrott and Lemieux [40]	Cross-sectional Telephone survey	Farmers, 100% males, *n* = 448	37.5 years	Not reported	Not reported	*Psychological*: knowledge about skin cancer *Social: *families' expectations and familial information giving	Not reported

Pichon et al.[6]	Cross-sectional Self-administered survey	Postal workers, 68% males, 53.63% White, *n* = 2,543	4 hours/day 12 years	Sunscreen (14–30%), wide-brim hat (20–34%), and sunglasses use (44%–63%)	Not reported	*Demographic: *ethnicity, sensitive skin type, female gender (sunscreen), and male gender (hat)	Self-identified race Social desirability bias

Salas et al. [41]	Cross-sectional Interview and observation Self-administered survey	Farmworkers, 100% males, 100% Latino, *n* = 326	14.23 years	Long-sleeved shirt (89.7%), wide-brimmed hat (6%), sunglasses (2.6%), and sunscreen (1.6%)	Not reported	*Demographic: *longer years of work	Design of the study Convenience sample Social desirability bias

Lewis et al. [33]	Cross-sectional Field observation Self-administered survey	Postal workers, 69% males, 51.3% White, *n* = 2,600	3.9 hours/work day 3.3 hours/nonwork day	Sunscreen during work (25%) Sunscreen during leisure time (12%) Hat during work (24%) Hat during leisure time (4%)	Not reported	*Demographic: *ethnicity, sun sensitive skin type, hours spent outdoors, female gender with sunscreen use, male gender with hat use, and family history of skin cancer	Design of the study Differences in time period for reporting work days (5 days) and nonworking days (2 days) sun safety behavior

Hammond et al. [42]	Cross-sectional Self-administered survey Sun protection chart diary	Horticulture, roading, and building, 82% males, *n* = 74	Not reported	Not reported	Not reported	*Psychological*: suntan attitude and perceived risk of skin cancer *Social: *workplace support	Validity and reliability was not checked Small sample size Convenience sample Low response rate

Gies et al. [43]	Cross-sectional Field observation Self-administered survey 4-day diary	Lifeguards, 59.3% females, *n* = 168	4.29 hours/day	Phoenix: sunglasses (90.4%) and sunscreen (76.4%) Austin: hat (37%) and shade (31.2%) Portland: shirt (31.3%)	Not reported	Not reported	2-day UVR exposure measurements Difference in availability of natural shade at each pool site

McCool et al. [34]	Cross-sectional Self-administered survey	General outdoor workers, *n* = 1,283	Not reported	Not reported	Not reported	*Demographic: *female gender, increased age, and higher education *Psychological*: knowledge, perceived priority of sun protection, and higher concern about sun exposure *Social: *workplace support	Use of other sun protection measures (e.g., clothing and hat) were not investigated Self-selection bias

Madgwick et al. [44]	Cross-sectional Self-administered survey	Construction workers, 100% male, *n* = 360	6.6 hours/day	Sunscreen (60%), wearing long-sleeved loose fitted tops and trousers (51%), sunglasses (44%), and wide-brimmed hat (23%)	Not reported	*Demographic: *age, personal history of skin cancer, family history of skin cancer, hours spent outdoors, and receipt of sun safety training *Psychological*: desire for suntan	Self-selection bias Response bias
